# Genetic Stability Developed for β-Carotene Synthesis in BR29 Rice Line Using Dihaploid Homozygosity

**DOI:** 10.1371/journal.pone.0100212

**Published:** 2014-06-17

**Authors:** Karabi Datta, Gayetri Sahoo, Sellappan Krishnan, Moumita Ganguly, Swapan K. Datta

**Affiliations:** 1 Department of Botany, Plant Molecular Biology and Biotechnology Laboratory, University of Calcutta, Kolkata, India; 2 Central Rice Research Institute, Cuttack, Odisha, India; 3 Department of Botany, Goa University, Goa, India; 4 Divisions of Crop Science, Indian Council of Agricultural Research (ICAR), Krishi Bhawan, New Delhi, India; Institute of Genetics and Developmental Biology, Chinese Academy of Sciences, China

## Abstract

Obtaining transgenic crop lines with stable levels of carotenoids is highly desirable. We addressed this issue by employing the anther culture technique to develop dihaploid lines containing genes involved in β-carotene metabolism. First, we used *Agrobacterium-* mediated transformation to develop primary transgenic plants containing the β-carotene biosynthetic genes, *phytoene synthase* (*psy*) and *phytoene desaturase* (*crtI*), which were engineered for expression and accumulation in the endosperm. Transgenic plants were recovered by selecting for the expression of the *phosphomannose isomerase* (*pmi*) gene. Dihaploid plants in addition to haploid and tetraploid plant were generated from anther cultures of these primary transgenic plants. In addition to anatomical features of stomata, pollen of different ploidy-plants, molecular analyses confirmed the stable integration of the genes in the anther culture-derived dihaploid plants, and the yellow color of the polished seeds indicated the accumulation of carotenoids in the endosperm. High performance liquid chromatography (HPLC) analysis of the carotenoid extract further confirmed the levels of β–carotene accumulation in the endosperms of the transgenic dihaploid rice seeds.

## Introduction

As a dominant cereal crop, rice serves as the staple food for more than half the world’s population. In Asian countries, rice accounts for 30–72% of the energy intake of the people. However, micronutrient concentrations in rice are not sufficient to meet the recommended daily dietary allowances (RDA) to sustain good health and minimize the occurrence of diet-related chronic diseases. Because of high per capita consumption of rice, increasing its nutritive value may have a significant positive impact on the health of the rice consuming population. Transgenic methods are therefore one option for improving the micronutrient content of rice [1]–[2].

Vitamin A deficiency, which affects the rod cells of the retina, proper immune system function and many other physiological functions in the human body, is a major nutritional problem facing Tropical Asia. Nearly 124 million children worldwide are deficient in vitamin A. Mammals cannot synthesize vitamin A themselves, and dietary vitamin A comes primarily from plants in the form of β–carotene and other provitamins [3].

In plants, carotenoids are present in all photosynthetic green tissues. The plant enzymes for carotenoid metabolism are encoded by the nuclear genome, and targeted to the plastids. In the plastids of green tissues, geranyl geranyl pyrophosphate, which is the biosynthetic precursor of β–carotene, is first converted to phytoene by the enzyme *phytoene synthase (psy*). In bacteria, *phytoene desaturase* (*crtI*) is the only enzyme responsible for converting phytoene to lycopene. The conversion of lycopene to β–carotene is then catalyzed by *lycopene cyclase*, which is not rate limiting in plants. Both the plant and microbial genes encoding the enzymes necessary for synthesizing β–carotene have been cloned, and their functions have been elucidated [4]–[7]. The overexpression of these genes to modify the amounts and types of carotenoids produced by plants particularly in their storage organs has been reported in food plants, such as carrot roots [8], tomato fruits [9], canola seeds [10], and potato tubers [11].

Extensive efforts to screen the germplasm of exotic rice to identify rice cultivars that accumulate considerable amount of carotenoids in the edible endosperm tissues are currently underway, and a few of these cultivars show the presence of a small amount of carotenoids in their unpolished seeds [2]. However, like other green plants, rice plants are able to produce β–carotene in their green tissues but do not typically accumulate it in non-green storage tissues, such as the edible endosperm. As an alternative approach, genetic engineering has been used effectively to synthesize β–carotene within the target tissue of rice endosperm [12]–[13].

Anther culture is regarded as a key technique for the induction of haploids in crop improvement efforts. The main advantage offered by the use of haploids is the rapid and complete homozygosity of the offspring, which allows for the easy selection of phenotypes for quantitative characteristics. Doubled haploid (DH) lines are also considered useful for genetic analysis. The first transgenic dihaploid indica rice line was generated via protoplast transformation using microspore-derived dihaploid calli [14]. Anther cultures of primary transgenics, can also be used to develop doubled haploids in less than a year [15].

In this study, we used *Agrobacterium*-mediated transformation to introduce a combination of transgenes, namely, *phytoene synthase* (*psy*) driven by the glutelin promoter and the bacterial *phytoene desaturase* (*crtI*) fused to the transit peptide sequence from the pea-Rubisco subunit driven by the CaMV35S promoter, for the biosynthesis of provitamin A in the endosperm. The Positech genetic selection marker system with the *phosphomannose isomerase* (*pmi)* gene served as an effective alternative to the use of antibiotic resistance or herbicide tolerance marker genes. To achieve stable expression in the minimum possible time frame, we generated anther cultures using these primary transgenic plants. The stable integration of the transgenes in the different anther culture-derived plants was confirmed by Southern blot analysis. RT-PCR analyses, combined with the characteristic yellow color of endosperm, revealed the gene expression levels. Spectrophotometry and HPLC were used to assess the total amount and overall spectrum of carotenoids that were produced in the transgenic double haploid lines.

## Materials and Methods

### Vector and Transformation

The vector pCaCar (obtained from the University of Freiburg, Germany) was used to perform the *Agrobacterium tumefaciens*-mediated transformation. The construct combines the daffodil (*Narcissus* p*seudonarcissus*) *psy* gene [16], driven by the endosperm-specific Gt1 promoter, with the bacterial (*Erwinia uredovora*) *desaturase* gene *crtI,* fused to the pea Rubisco small subunit transit peptide sequence [5] and under the control of the constitutive 35S (CaMV) promoter. The *phosphomannose isomerase* (*pmi*) gene, driven by the 35S (CaMV) promoter, as selectable marker gene was present in the same vector [3].

Embryogenic calli derived from the immature embryos of rice cultivar BR29 were inoculated with *A. tumefaciens* strain EHA 101. The callus transformation procedure has been described previously [3]. For Positech selection, we used 1.5% (w/v) mannose with 2.0% (w/v) sucrose for the first selection, 2.0% (w/v) mannose with 1.5% (w/v) sucrose for the second selection and 2.5 (w/v) mannose with 1% (w/v) sucrose for the third selection. Regeneration and rooting were performed as described previously [17]. Mannose-resistant rice plants were grown in a containment greenhouse, following a day/night temperature regime of 29^o^/22±2°C with 70–85% relative humidity.

### Polymerase Chain Reaction (PCR) and Southern Blot Analysis

Genomic DNA was isolated from frozen leaves of 1-month-old rice plants, and 100 µg of template was used for PCR analysis. The gene-specific primer used was described previously [13]. For Southern blot analysis, plant genomic DNA was extracted from fresh leaves of transgenic and nontransgenic control plants. Next, 10 µg of DNA was digested using restriction endonucleases, *EcoR*I for *crtI*, and *Xho*I for *pmi* gene (Invitrogen, CA) and the digested DNA was separated on a 1% (w/v) TAE-agarose gel. Southern membrane transfer, hybridization and autoradiography were all performed as described previously [17].

### Anther Culture

The spikes and ensheathing leaves from the middle of the panicles of transgenic BR-29 plants grown in containment were removed and used for the experiments [18]. The selected spikes were surface sterilized in 70% ethanol, for 30 s, rinsed thoroughly in sterile distilled water and dried over blotting paper. Florets were tapped lightly against the edge of a petri dish, to release the anthers into the callus induction medium (N6 medium containing 50 g l^−1^ Maltose, 2 mg l^−1^ 2, 4-D, and 2 mg l^−1^ Kinetin and supplemented with 10 mg l^−1^ thiamine HCl, 300 mg l^−1^ Casein hydroxylate, 300 mg l^−1^ glutamine and 8 g l ^−1^ agar, pH 5.8). Dividing microspores become visible after 6−8 d of culture and callus induction becomes visible after 6–7 weeks of culture in the dark at 28°C. The calli were transferred to regeneration medium (N6 medium containing 60 g l^−1^ Maltose, 2 mg l^−1^ Kinetin, 0.5 mg l^−1^ NAA, and 0.5 mg l^−1^ IAA and supplemented with 500 mgl^−1^ Proline, and 500 mgl^−1^ Casein hydroxylate and 8 g l^−1^ agar, pH 5.8). The cultures were incubated for 3–4 weeks with a 16 h photoperiod of 5000 lux intensity at 28°C ±1°C. The plants were transferred to MS medium without hormones to induce rooting and then transferred to the greenhouse.

### Leaf Anatomy and Stomatal Structure

For leaf anatomical studies, free-hand vertical sections (vs) of the leaves of different types (haploid, dihaploid, or tetraploid) of anther culture-derived rice plants were stained with safranin and photographed using a Carl Zeiss Axioplan-2 microscope equipped with an automatic exposure system. Epidermal peels were obtained from fresh leaf materials following the standard method [19] for stomatal study. Briefly, 1-cm-long pieces of the collected leaves were scraped on their abaxial sides to remove most of the cells above the adaxial epidermis, and the isolated adaxial epidermis was then stained with 1% safranin for 30 s, washed thoroughly in distilled water, mounted with diluted glycerine and photographed using a Carl Zeiss Axioplan-2 microscope. All photographs were taken at a similar magnification (×1800).

### RT-PCR

RNA was isolated from the polished seeds of transgenic and non-transgenic control plants. Plant samples were powdered in liquid nitrogen, and total RNA was extracted using the RNAeasy extraction kit (Qiagen, Germany). RT-PCR was performed with a total of 2 µg of RNA using the Qiagen one-step RT-PCR kit with a gene-specific primer. The RT-PCR products were resolved on a 1.2% TAE-agarose gel.

### Carotenoid Extraction and HPLC Analysis

Dehusked seeds from transgenic and non-transgenic plants were polished for 10 h at 200 rpm in petri dishes containing emery paper. Polished seeds were ground to a fine powder using the cyclone sample mill (Udy Co., USA), and 0.5 g of seed powder was used for acetone (2 ml) extraction. After extracting twice with acetone, a ½ volume of petroleum ether was added to the supernatant. Phase separation was then performed by adding water. The colored top layer of carotenoids was dried, and the dried extract was re-dissolved in petroleum ether and a spectrophotometric reading was taken at 453 nm.

The distribution of carotenoids was determined by HPLC. Dried extract was dissolved in acetone, and 40 µl of acetone extract was applied to HPLC for peak separation. HPLC was performed using a Waters Alliance 2690 separation module (Waters Corporation, Milford, MA, USA), equipped with a Waters 996 photodiode array detector and Water Millennium^32^ Chromatography Manager. Samples were separated on a Waters YMC Carotenoids column (4.6×250 mm, 5 µm), after passing through a guard column of the same material (4.0×10 mm, 5 µm), and eluted with solvent system A (acetonitrile: tetrahydrofuran: Water::: 10∶4∶6) and solvent system B (acetonitrile: tetrahydrofuran: Water::: 10∶8.8∶1.2). The column was developed with 100% A for the first 3 min, followed by a linear gradient to 100% B over 7 min and subsequently remained at B for 20 min. The column was equilibrated for 10 min between samples.

## Results

### Transformation

A large number of putative transgenic plants containing *psy* and *crtI* were obtained using the Positech genetic selection system with *phosphomannose isomerase* (*pmi)* as the marker gene. A total of 278 plants were found to be positive based on PCR and Southern analysis. Different plants showed different patterns of integration ranging from single to multiple copy number.

### Anther Culture

A total of 790 green plants were obtained from anther cultures of the boots collected from 23 selected plants that were positive for both the *psy* and *crtI* genes. Of those green plants, 390 were found to be positive, when primarily checked by PCR analysis. Different phenotypic variations were identified in the anther culture-derived plants (Table 1).

**Table 1:** Status of Anther Culture-derived Transgenic β-carotene.

Plant line	No of green plantsgenerated	No of transgenicplants	No of haploidplants (PCR ^+^)	No of abnormalplants (PCR^+^)	No of fertileplants (PCR ^+^)
**ACSKBR-13**	61	29	16	8	5
**ACSKBR-28**	4	2	0	0	2
**ACSKBR-29**	133	75	39	11	25
**ACSKBR-32**	19	10	5	0	5
**ACSKBR-33**	19	8	3	3	2
**ACSKBR-35**	43	12	7	2	3
**ACSKBR-40**	10	6	0	5	1
**ACSKBR-41**	57	32	11	15	6
**ACSKBR-44**	31	14	5	2	7
**ACSKBR-45**	16	6	2	2	2
**ACSKBR-46**	55	27	10	10	7
**ACSKBR-48**	24	9	4	2	3
**ACSKBR-49**	4	3	2	1	0
**ACSKBR-50**	13	10	6	0	4
**ACSKBR-51**	22	10	4	2	4
**ACSKBR-52**	8	5	1	0	4
**ACSKBR-54**	9	9	3	2	4
**ACSKBR-56**	17	1	1	0	0
**ACSKBR-57**	11	2	0	0	2
**ACSKBR-58**	17	14	7	5	2
**ACSKBR-59**	46	31	15	7	9
**ACSKBR-62**	43	18	14	2	2
**ACSKBR-64**	128	57	27	18	12
**Total**	**790**	**390**	**182**	**97**	**111**

Different parameters considered like No. of plants generated, No. of PCR positive transgenic plants, No. of PCR positive haploid plants, No. of PCR positive abnormal plants, No. of PCR positive fertile plants of anther culture derived transgenic β-carotene plants.

**Figure 1:**
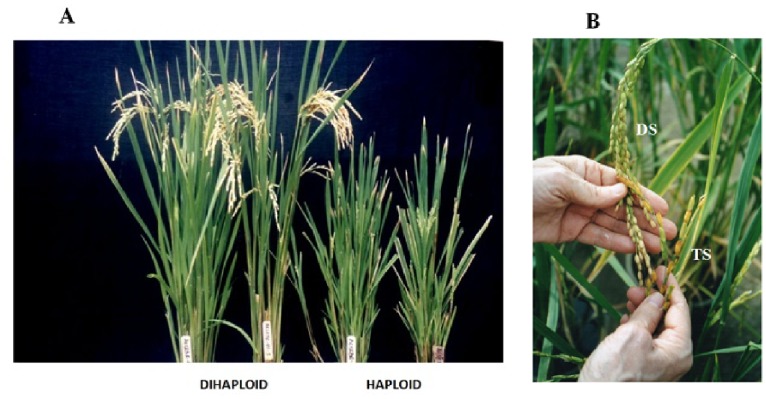
Phenotypic variation of anther culture-derived transgenic plants. (A) Dihaploids showed normal phenotypes with fertile seeds, the LTB haploids were sterile and exhibited stunted growth (B) Dihaploid plants showed normal sized seeds whereas the LTB tetraploids showed larger sized seeds. DS-Dihaploid seeds; TS- LTB Tetraploid seeds.

**Figure 2:**
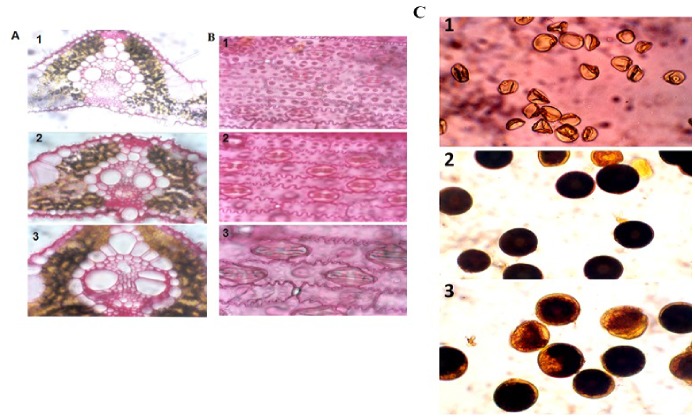
Vertical sections of leaves (A) with corresponding stomatal structures (B) and pollen viability (C) of selected haploid (A1, B1, C1), dihaploid (A2, B2, C2), and tetraploid (A3, B3, C3) plants. LTB Haploids have smaller stomata and are sterile, dihaploids have normal stomata and are fertile and LTB tetraploids have bigger stomata with mostly non-fertile pollen. All photographs were taken at a similar magnification (x1800).

**Figure 3:**
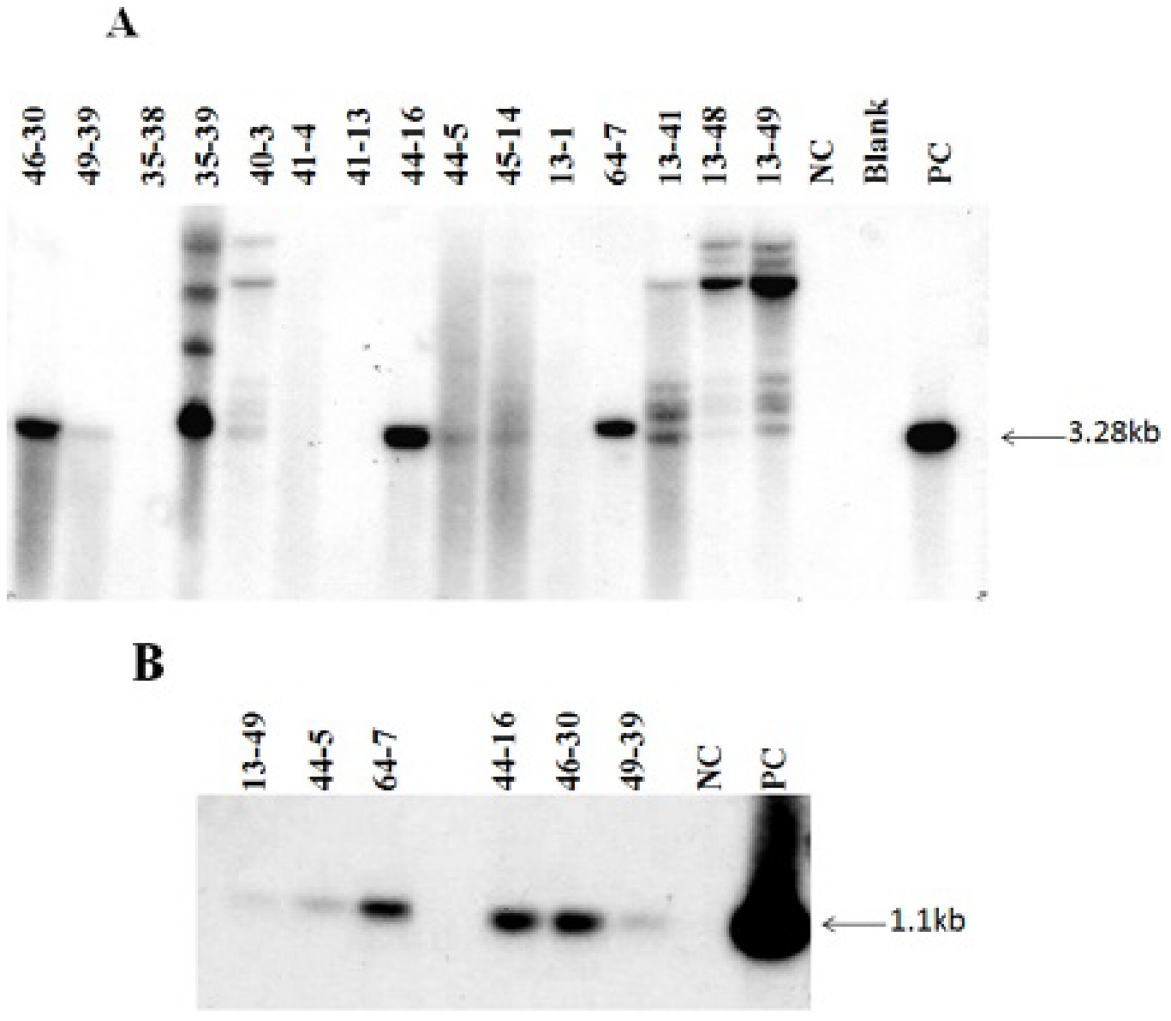
Southern blot analysis showing integration of the *crtI* (3.28 kb) and *pmi* (1.1 kb) gene in anther culture-derived plants. Stable integration of 3.28 kb *EcoR*I restriction fragment corresponding to the *crtI* gene and 1.1 kb *Xho*I restriction fragment corresponding to the *pmi* gene in different plant lines. No hybridization signal was observed in non-transgenic control plants. 10 µg of DNA was digested and it was separated on a 1% TAE-agarose gel.

The phenotypic variability of the anther culture-derived plants is shown in Fig. 1A and 1B. Variations in leaf anatomical features, stomatal size and pollen morphology and viability were observed in the three different types of plants [likely to be (LTB) haploid, dihaploid, and likely to be (LTB) tetraploid]. The number of mesophyll cell layers, the size of the vascular bundle (Fig. 2A), the size of stomata (Fig. 2B) and pollen characteristics (Fig. 2C) were found to differ due to changes in the ploidy level, although the number of chromosomes in plants with different phenotypes was not determined. Based on their phenotypic traits, a total of 111 doubled haploids, i.e., normal phenotype, were found to be fertile in Fig. 2C-2 (all pollen obtained from dihaploid plants seems to be fertile). By contrast, staining analyses revealed that all pollen from the smaller bushy plants was sterile (Fig. 2C-1), indicating likely to be (LTB) haploid plants. The bigger pollen from the broader-leaved plants (not shown) with bigger stomata and cells size as shown in Fig. 2A-3 & 2B-3 with mostly non-fertile pollen, likely to be (LTB) tetraploid plants (Fig. 2C-3) and with larger seed (Fig. 1B).

### Integration of the Transgenes

Insertion of the transgenes into the genome of the doubled haploids (normal type) was confirmed by PCR and Southern blot analysis. The presence of 3.28 kb and 1.1 kb band upon digestion with *EcoR*I and *Xho*I confirmed the integration of the *crtI and pmi* gene respectively. Independent transformants contained one to several rearranged transgene copies (Fig. 3A and 3B).

### Expression

**Figure 4:**
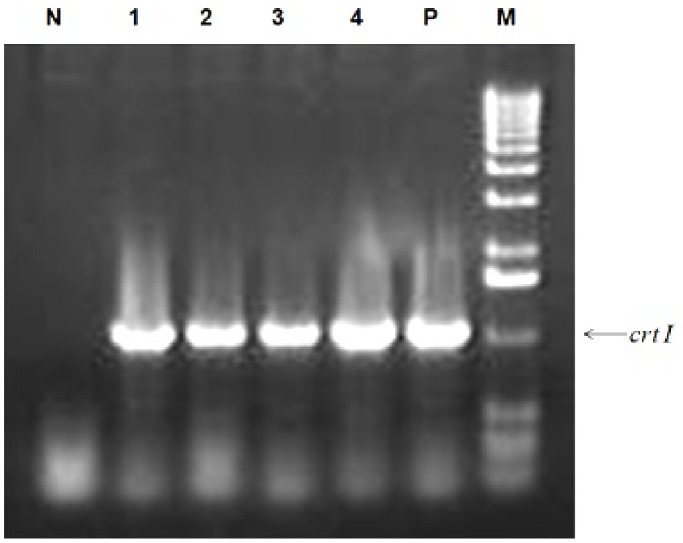
RT-PCR showing the expression of *crtI* mRNA in anther culture-derived plants. Expression analysis of the *crtI* gene in homozygous anther culture-derived transgenic plant line. RT-PCR was performed with 2 µg of RNA and products were resolved on a 1.2% TAE-agarose gel. ‘N’ represents the Negative Control, ‘P’ represents the Positive Control and ‘M’ represents the Molecular marker.

Reverse transcription polymerase chain reaction (RT-PCR) confirmed the expression of *crtI* in seeds, based on the presence of the expected 1.03 kb amplicon for *crtI* cDNA, which is absent in the non-transgenic control plants (Fig. 4).

**Figure 5:**
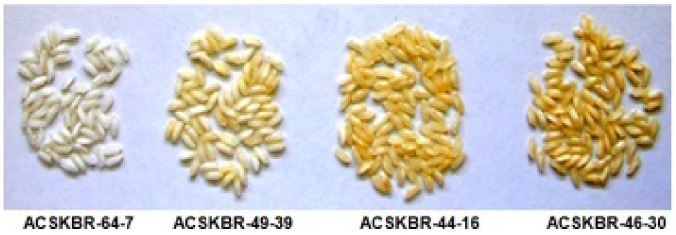
Yellow endosperms from the polished grains. Variations in yellow coloration from carotenoids in transgenic double haploid rice plants showing different levels of expression. Variations in carotenoid content was found in different transgenic lines like ACSKBR-46-30 showed 3.188 µg g^−1^, ACSKBR-44-16 showed 3.036 µg g^−1^, ACSKBR-49-39 showed 2.12 µg g^−1^ whereas ACSKBR-64-7 showed 0.257 µg g^−1.^

**Figure 6:**
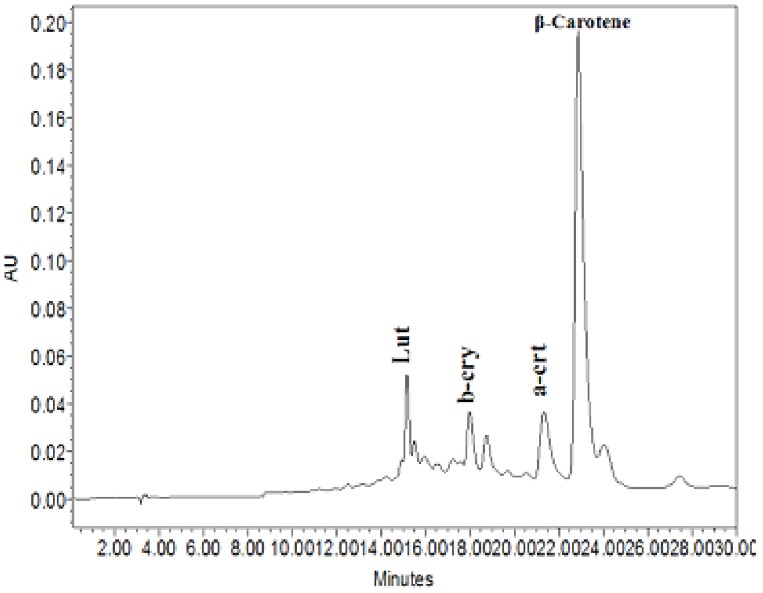
HPLC of homozygous transgenic rice lines. HPLC chromatogram of AC SKBR- 46–30 showing the β-carotene peak together with other carotenoid peaks (Lut-Lutein, b-cry-b-cryptoxanthin, a-crt- a-carotene) in the carotenoid extract of polished yellow seeds from doubled haploid transgenic plants. 0.5 g of seed powder was used for acetone (2 ml) extraction and spectrophotometric reading was taken at 453 nm.

Mature seeds from individual transgenic plants derived from anther cultures of the individual primary transgenic lines were polished together with seeds from non-transgenic control plants. Variations in the carotenoids levels and intensity of yellow coloration indicate the differential expression of transgene and carotenoid accumulation in the endosperms of the individual plants (Fig. 5). Spectrophotometric and HPLC analyses were used to quantify the total carotenoids and β–carotene levels (Fig. 6). The total carotenoid levels ranged from 0.257 µg g^−1^ to 3.188 µg g^−1^ in the endosperms of individual double haploid plants. Seeds from individual plants derived from the same primary transgenic plant showed different levels of carotenoids and β–carotene content.

## Discussion

The development of stable homozygous lines is a primary prerequisite for the evaluation and selection of transgenic lines and for use in cross-breeding programs to transfer the transgenes into other backgrounds. Normally when, using a transgene as a marker, the selection of homozygous lines requires more than three generations, depending on the copy number and position of the inserted genes. The anther culture technique reduces the breeding cycle and rapidly fixes homozygosity via the production of doubled haploids [20]. In our study, we have carried out the anther culture of primary transgenic plants to generate stable, doubled haploid rice producing β–carotene within the shortest possible time. Several factors, such as the quality of the donor plants, genotypic variations, media composition and the handling of the cultures, play a crucial role in plant regeneration from anther cultures [21].

Previously, we reported the bioengineering of indica rice cultivars expressing the β–carotene metabolic pathways in the endosperm via the biolistic method of transformation, wherein three different plasmid vectors were delivered together by co-transformation [13]. To avoid complex integration patterns of the inserted genes, in the present study we used *Agrobacterium-*mediated transformation with the pCaCar vector, which contains the same three genes in a single cassette.

There are two ways to obtain dihaploid transgenic plants. The first is to develop anther culture-derived dihaploid (pollen-derived) embryogenic cell lines that can be used for transformation, i.e., dihaploid cell lines are developed first. The second approach is to first develop transgenic plants (heterozygous lines) containing the gene of interest and then use the haploid pollen from these transgenic lines, to develop dihaploid plants. Although, we observed sizeable phenotypic variability in the anther culture-derived plants as shown in Table 1, a good number of fertile dihaploid transgenic plants was obtained.

Under culture conditions, the chromosomes of anther culture-derived plants undergo spontaneous duplication [18]. The application of colchicines may also be useful in some cases. We report here, the regeneration of fertile β-carotene producing dihaploid plants which occurs via direct embryogenesis involving the spontaneous doubling of the chromosomes.

Southern blot analysis confirmed the integration of *crtI* gene. Integration of *pmi* gene was also checked with the transgenic lines which showed simple pattern of gene integration. Carotenoid expression was found to vary, as observed by spectrophotometric and HPLC analyses of polished seeds derived from different individual plants. The seeds obtained from individual anther culture-derived plants from the same primary transgenic plant also exhibited variations in the level of carotenoid accumulation, which could be due to differences in the copy number of the inserted genes in the individual primary transgenic lines and subsequent differences in segregation patterns during meiosis. This process is also thought to produce similar variations in the expression levels of carotenoids that have been noted previously in the transgenic progeny of heterozygous mother plants [3]. Carotenoid profile of transgenic seeds showed the presence of β-carotene, lutein, β-cryptoxanthin and α-carotene. Different pattern of gene integration also effect the levels of carotenoid as also previously demonstrated [3]. Variations in carotenoid content was found in different transgenic lines like ACSKBR-46-30 showed 3.188 µg g^−1^, ACSKBR-44-16 showed 3.036 µg g^−1^, ACSKBR-49-39 showed 2.12 µg g^−1^, ACSKBR-64-7 showed 0.257 µg g^−1^. These variation in carotenoid content caused differences in seed colorations.

Carotenoid biosynthesis occurs on the membranes of chloroplasts, chromoplasts and amyloplasts, which are genetically identical plastids with very different internal membrane architectures. Despite ongoing research into the biosynthesis and accumulation of carotenoids in model organisms, information on the regulation of the biosynthetic pathways operating in plants of agronomic importance is limited. Due to the insufficient understanding of how metabolic assembly is controlled in plastids with different membrane architectures, efforts to improve and stabilization the carotenoid level and nutrition profile composition in cereal crops using DH lines may support accelerating the molecular breeding of nutrition enriched crop improvement.
